# Synthesis and Biological Evaluation of New Ligustrazine Derivatives as Anti-Tumor Agents

**DOI:** 10.3390/molecules17054972

**Published:** 2012-04-30

**Authors:** Penglong Wang, Gaimei She, Yanan Yang, Qiang Li, Honggui Zhang, Jie Liu, Yinqiu Cao, Xin Xu, Haimin Lei

**Affiliations:** 1 School of Chinese Pharmacy, Beijing University of Chinese Medicine, Beijing 100102, China; 2 Institute of Materia Medica, Chinese Academy of Medical Sciences Peking Union Medical College, Beijing 100050, China

**Keywords:** angiogenesis, anti-tumor, combination principles, ligustrazine derivatives, low toxicity

## Abstract

To discover new anti-cancer agents with multi-effect and low toxicity, a series of ligustrazine derivatives were synthesized using several effective anti-tumor ingredients of Shiquandabu Wan as starting materials. Our idea was enlightened by the “combination principle” in drug discovery. The ligustrazine derivatives’ anti-tumor activities were evaluated on the HCT-8, Bel-7402, BGC-823, A-549 and A2780 human cancer cell lines. In addition the angiogenesis activities were valued by the chick chorioallantoic membrane (CAM) assay. 1,7-bis(4-(3,5,6-Trimethylpyrazin-2-yl)-3-methoxyphenyl)-1,6-heptadiene-3,5-dione (**4**) and 3 α,12 α-dihydroxy-5β-dholanic acid-3,5,6-trimethylpyrazin-2-methyl ester (**5**) not only displayed antiproliferative activities on these cancer cells, but also dramatically suppressed normal angiogenesis in CAM. The LD_50_ value of the compound **5** exceeded 3.0 g/kg by oral administration in mice.

## 1. Introduction

Traditional Chinese Medicine (TCM) uses multi-target effects to potentiate synergism of multi-effective compounds [1,2,3]. Due to its positive therapeutic effects, low toxicity and minimal side effects, TCM is increasingly accepted around the World [4,5,6]. As an application of the structure combination idea, researchers have recently explored a novel field of discovering lead compounds from TCMs. Zhang chose the major bioactive components in one classic TCM preparation to synthesize five novel esters connecting verticinone with bile acids, which showed satisfactorily effects and could be used as antitussive and expectorant agents in the future [7,8]. We chose brain protective ingredients from one TCM recipe to synthesize ligustrazine-protocatechuic acid (TP, C_16_H_23_N_2_O_5_). TP exhibited synergistic pharmacodynamic effects compared with the ligustrazine alone, protocatechuic acid alone and the mixture of ligustrazine and protocatechuic acid [9].

Shiquandabu Wan, combining *Rhizoma Curcumae Longae* and *Calculus bovis*, is a classic TCM recipe used to treat cancer [10,11]. Many anti-tumor ingredients were found in this recipe, including ligustrazine (TMP, C_8_H_12_N_2_), curcumin (CU, C_21_H_20_O_6_), deoxycholic acid (DA, C_24_H_40_O_4_), cholic acid (CA, C_24_H_40_O_5_), oleanolic acid (OA, C_30_H_48_O_3_), cinnamic acid (CIA, C_9_H_8_O_2_), glycyrrhetinic acid (GA, C_30_H_46_O_4_), pachymic acid (PA, C_33_H_52_O_5_) and dehydropachymic acid (DPA, C_33_H_50_O_5_) [12,13,14,15,16,17,18,19,20,21]. TMP could not only be rapidly absorbed into blood, but also pass through the blood-brain barrier and blood-labyrinth barrier [22]. If coupled with TMP, the target compounds could be convenient for administration and permeable to the physical barriers. Bile acids played an important role in the bile acid-positive cancer cells and improving the tumor-targeting chemotherapeutic effects on liver or colon cancers [23]. As a part of our combination-idea on the use of classic TCM preparations to discover new lead compounds, we selected those anti-tumor ingredients as starting materials to synthesize TMP derivatives [24,25]. These compounds’ anti-tumor activities were evaluated on the HCT-8, Bel-7402, BGC-823, A-549 and A2780 human cancer cell lines by MTT assay, and these compounds’ angiogenesis activities in the chick chorioallantoic membrane (CAM) assay were also evaluated.

## 2. Results and Discussion

### 2.1. Chemistry

**Scheme 1:**
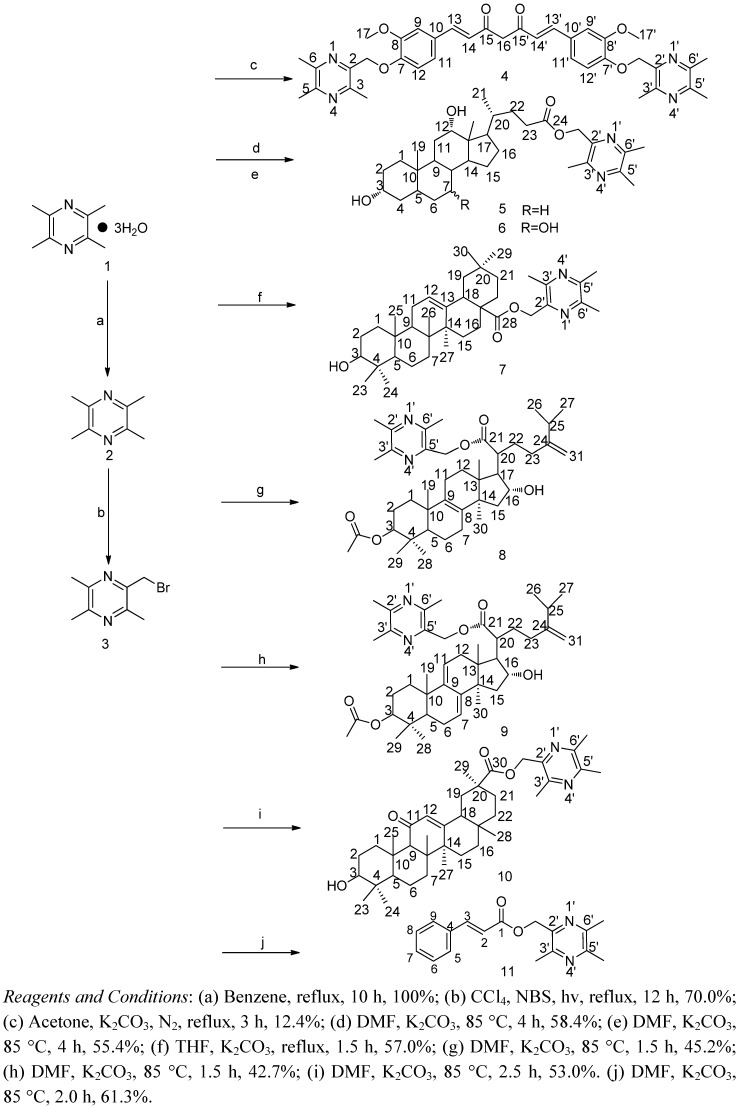
Synthesis routes to ligustrazine derivatives.

Compound **3** was synthesized from TMP and *N*-bromosuccinimide (NBS) via free radical reaction. The typical subsequent synthetic procedure involved the combination of bromo TMP and other starting materials through the formation of ester or ether bonds under alkaline condition (as shown in Scheme 1). Compound **3** and CU were dissolved in dry acetone and refluxed for 3 h with N_2_ protection to give compound **4**. CU is a diarylheptanoid having phenyl rings at 1,7 positions of *n*-heptane and it is unstable under alkaline conditions, so the yield of **4** is low. Treatment of compound **3** with DA in dry dimethylformamide (DMF) at 85 °C for 4 h, afforded compound **5**. Similarly, we obtained compounds **6**, **8**, **9**, **10** and **11** by coupling compound **3** with CA, GA and CIA, respectively. Compound **7** was obtained from compound **3** and OA in dry tetrahydrofuran (THF) after refluxing for 1.5 h. Of all the compounds, **4**, **5**, **6**, **7**, **8**, **9** and **10** were new compounds and **11** had been reported, although compound **11**’s antitumor activities had not been explored [26]. 

The structures of all the target compounds were elucidated by HRMS and NMR spectroscopy. The ^1^H-NMR and ^13^C-NMR data of compound **4** was symmetric, as its structure is highly symmetric. Because of the formation of hydrogen bonds with the neighbouring carbonyl groups (CO), the two signals at *δ* 16.03 and *δ* 5.80 in the ^1^H-NMR correspond to the H16 and H16' protons.

The difference between **8** and **9 **is that **8** lacks an olefinic bond in the parent nucleus structure. According to reference [27], we observed a small difference in the NMR data, *δ* 0.58 (H-18), 5.45 (H-7) and 5.25 (H-11) in ^1^H-NMR and* δ* 120.7 [C(7)] and 116.1 [C(11)] in ^13^C-NMR are characteristic peaks of compound **9**, while *δ* 0.71 (H-18) in ^1^H-NMR and *δ* 134.2 [C(8)] and 134.3 [C(9)] in ^13^C-NMR are characteristic peaks of compound **8**. Furthermore, HRMS (ESI) gave all the molecular ion peaks corresponding to the molecular weights of the confirmed target compounds. 

### 2.2. Biological Activities

#### 2.2.1. MTT Test

The anti-proliferation effects of the TMP derivatives and reference compounds were evaluated in different tumor cells using the MTT assay. As shown in Table 1, after combination with TMP, most of the synthesized compounds have better anti-tumor activities than those of the starting materials. Compounds **4** and **5** exhibited much better anti-tumor activities than the references materials on all cell lines. It has been noticed that compounds **5** and **6** have similar structures and the compound **5** shows much better anti-tumor activities against the five cell lines than compound **6**. This manifests some structure-activity relationship patterns suggesting that the 7-position substituents is the key functional group to decrease the anti-tumor activities. In addition, both compounds **9** and **11** exhibit potent activities against the A549 cell line with IC_50_ values of 8.770 μg/mL and 7.833 μg/mL, respectively. Compounds **7** (IC_50_ 7.611 μg/mL) and **11** (IC_50_ 9.400 μg/mL) have similar activities against Bel-7402 cell. Moreover, compound **7** shows activity against HCT-8 cell line with an IC_50_ value of 9.273 μg/mL.

**Table 1:** Anti-proliferative effects of ligustrazine derivatives and compared materials.

Compound	IC_50_ (μg/mL)				
	Bel 7402	A549	HCT-8	BGC-823	A2780
TMP, DA, CA, OA, GA, CIA, PA and DPA	— ^a^	—	—	—	—
CU	6.496	6.521	6.278	6.806	6.520
**4**	6.391	5.890	7.106	5.472	5.540
**5**	8.012	6.688	7.426	6.660	6.619
**6**	—	—	—	—	—
**7**	7.611	—	9.273	—	—
**8 **	—	—	—	—	—
**9**	—	8.770	—	—	—
**10**	—	—	—	—	—
**11**	9.400	7.833	—	—	—

^a^ IC_50_ > 10.0 ig/mL. We set a strict standard to the evaluation of anti-tumor activities. The maximal concentration of tested compounds is 10.0 ig/mL. When IC_50_ > 10.0 ig/mL, we considered the compounds’ anti-tumor activities were too weak to do further research. Worth mentioning is that CU, which has been recognized as a potential chemoprevetative and chemotherapeutic agent [14], is the standard in five cell lines test.

#### 2.2.2. Angiogenesis Activity

Anti-angiogenesis as a way of treating primary tumors and reducing their metastases had been proposed by Judah Folkman in 1971 [28]. Clinical practice has also proved that antiangiogenic drugs could enhance the treatment efficacy of cytotoxic chemotherapy [29]. Especially the multi-effective antitumor agents presented positive effects cancerous persons [30]. This was supported by the compound linifanib, which was a novel, orally active multi-targeted agent. Linifanib exhibited potent antitumor and antiangiogenic activities against a broad spectrum of experimental tumors and malignancies in patients [31]. According to the references, CU could inhibit angiogenesis [32], consequently the new compounds’ angiogenesis activities were evaluated by the chick chorioallantoic membrane (CAM) assay. 

**Figure 1:**
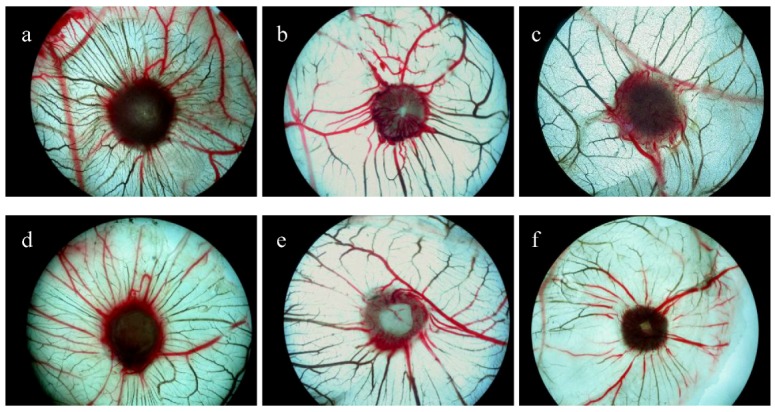
Microvascular proliferation of **4** and **5** on CAM (×50). (**a**) Control for compound **4** group. (**b**) 10 μg/egg for compound **4** group. (**c**) 40 μg/egg for compound **4** group. (**d**) Control for compound **5** group. (**e**) 10 μg/egg for compound **5** group. (**f**) 40 μg/egg for compound **5** group.

The model was established according to the reference and our previous work [33,34]. As shown in Figure 1 and Table 2, this study directly shows that all of the target compounds can inhibit the angiogenesis of the CAM. Especially compounds **4** and **5** dramatically block the growth of new vessels under both doses of 10 μg/egg and 40 μg/egg. The anti-angiogenesis activity of compound **6** is weaker than that of compound **5** in the CAM assay, in agreement with their anti-tumor activities. These prove that 7th-position is the functional group deciding its activity.

**Table 2:** Anti-angiogenesis effects of ligustrazine derivatives on CAM (mean ± s, n = 6).

Compound	Control ( 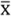 ± s)	Treatment ( 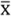 ± s)	Dose (μg/egg)
**4**	10.0 ± 2.19	3.8 ± 2.79 **	10
**4**	10.0 ± 2.19	1.4 ± 1.22 **	40
**5**	12.5 ± 2.59	4.8 ± 3.71 **	10
**5**	12.5 ± 2.59	7.5 ± 3.40 *	40
**6**	9.0 ± 2.68	6.2 ± 2.40	10
**6**	9.0 ± 2.68	5.7 ± 2.07	40
**7**	12.5 ± 2.59	12.0 ± 2.90	10
**7**	12.5 ± 2.59	9.5 ± 4.55	40
**8**	12.5 ± 2.59	9.7 ± 3.98	10
**8 **	12.5 ± 2.59	10.8 ± 5.12	40
**9**	8.67 ± 1.03	7.7 ± 2.16	10
**9**	8.67 ± 1.03	5.7 ± 1.21	40
**10**	10.3 ± 1.50	9.5 ± 1.76	10
**10**	10.3 ± 1.50	9.8 ± 2.14	40
**11**	17.7 ± 5.28	10.3 ± 2.73	10
**11**	17.7 ± 5.28	7.0 ± 3.03 *	40

* *P* < 0.05, ** *P* < 0.01 *vs.* control; control group: Physiological saline.

#### 2.2.3. Acute Toxicity

Oral therapeutic remedies are easy and convenient for cancer sufferers [35]. Both compounds **4** and **5** showed better antitumor activities, but unluckily the yield of compound **4** is low and CU is poorly available following oral administration to patients [36]. As one of the bile acids, DA could be reabsorbed and complete the enterohepatic circulation in the intestine with very little loss. Accordingly, compound **5** may have better oral absorbability than that of compound **4**, so we just evaluated the acute toxicity of compound **5** by gavage. During two weeks, there were no deaths or signs of toxicity observed after oral administration of the maximum tolerated dose (3 g/kg). Moreover, both phase I and phase II clinical trials indicated that CU was quite safe and exhibited therapeutic efficacy [37,38]. We infer that **4** could also have a low toxicity *in vivo*. 

## 3. Experimental

### 3.1. Chemistry

Reactions were monitored by TLC using silica gel coated aluminum sheets (Qingdao Haiyang Chemical Co., Qingdao, China) and visualized in UV light (254 nm). ^1^H-NMR and ^13^C-NMR assays were recorded on a BRUKER AVANCE 500 NMR spectrometer (Fällanden, Switzerland) and chemical shifts are reported in* δ* (ppm). Mass spectra were obtained by using Q-TOF and (ESI^+^) with an LC Autosampler Device: Waters 2695 instrument (New York, NY, USA). Melting points (uncorrected) were measured on an X-5 micro melting point apparatus (Beijing, China). Flash chromatography was performed using 300 mesh silica gel. The yields were calculated based on the last step reaction.

*2-(Bromomethyl)-3*,*5*,*6-trimethylpyrazine* (**3**). Compound **1** (TMP·3H_2_O, 25.080 g, 0.132 mol) was dissolved in benzene (70 mL). The mixture refluxed for 10 h to evaporate the water of crystallization and compound **2** was obtained. Compound **3** was prepared from compound **2 **(TMP, 19.992 g, 0.147 mol) and NBS (21.004 g, 0.118 mol) in refluxing carbon tetrachloride. The reaction mixture was illuminated by a 60W tungsten light bulb for 12 h. Then the mixture was filtered and the ﬁltrate was evaporated under vacuum, and the crude oil-product was obtained. Compound **3**, with 70% purity, was not purified further as it caused a strong mucous membrane irritation.

*1*,*7-bis(4-(3*,*5*,*6-Trimethylpyrazin-2-yl)-3-methoxyphenyl)-1*,*6-heptadiene-3*,*5-dione* (**4**). Compound **3** (0.660 g, 3.070 mmol) and CU (0.565 g, 1.535 mmol) were dissolved in dry acetone, then K_2_CO_3_ (0.700 g, 5.072 mmol) was added. The mixture was refluxed for 3 h with N_2_ protection, then it was filtered and the ﬁltrate was dried under vacuum. The product was separated by flash chromatography with petroleum ether-acetone (4:1) as eluent and recrystallized from acetone. Yellow crystals, m.p.: 206.9–207.7 °C, yield 12.4%. ^1^H-NMR (CDCl_3_) *δ* (ppm): 3.90 (s, 6H, H-17,17'), 16.03 (s, 1H, H-16), 5.80 (s, 1H, H-16), 6.51 (d, *J* = 16.0 Hz, 2H, H-14, 14'), 7.60 (d, *J* = 16.0 Hz, 2H, H-13, 13'), 7.06–7.13 (m, 6H, H-9, 11, 12, 9', 11', 12'); pyrazine ring: 5.26 (s, 4H, CH_2_-2, 2'), 2.63 (s, 6H, CH_3_-6, 6'), 2.53 (brs, 12H, CH_3_-3, 3', 5, 5'). ^13^C-NMR (CDCl_3_) *δ* (ppm): 150.1 [C(7, 7')], 149.9 [C(8, 8')], 110.4 [C(9, 9')], 128.7 [C(10, 10')], 122.3 [C(11, 11')], 113.9 [C(12, 12')], 140.3 [C(13, 13')], 122.3 [C(14, 14')], 183.2 [C(15, 15')], 101.4 [C(16, 16')], 56.0 [C(17, 17')]; pyrazine ring: 70.9 (2, 2'-CH_2_), 151.3 [C(2, 2')], 145.4 [C(3, 3')], 148.6 [C(5, 5')], 150.0 [C(6, 6')], 21.7 (6, 6'-CH_3_), 21.4 (5, 5'-CH_3_), 20.7 (3, 3'-CH_3_). HRMS (ESI) *m/z*: 637.5045 [M+H]^+^, calcd. for C_37_H_41_N_4_O_6_ 637.3026.

*3* *α*,*12α-Dihydroxy-5β-dholanic acid-3*,*5*,*6-trimethylpyrazin-2-methyl ester* (**5**). Compound **3** (1.402 g, 6.521 mmol) and DA (2.556 g, 6.521 mmol) were dissolved in dry DMF, then K_2_CO_3_ (2.503 g, 18.138 mmol) was added and the mixture was kept at 85 °C for 4 h. The warm reaction mixture was poured into ice-water and the crude product was extracted with ethyl acetate. After drying the organic layer over anhydrous Na_2_SO_4_ and evaporating the solvent under vacuum, the crude product was purified by flash chromatography with petroleum ether-ethyl acetate (4:1) as eluent. White amorphous solid, m.p.: 83.6–84.3 °C, yield 58.4%. ^1^H-NMR (CDCl_3_) *δ* (ppm): 0.67 (s, 3H, H-18), 0.92 (s, 3H, H-19), 0.97 (d, 3H, H-21), 3.62 (m, 1H, H-3), 3.97 (m, 1H, H-12), 5.21, 5.18 (ea, d, *J* = 12.5 Hz, 1H, CH_2_-2'), 2.54 (s, 3H, CH_3_-6'), 2.52 (s, 3H, CH_3_-5'), 2.51 (s, 3H, CH_3_-3'), 1.00–2.50 (28H, methyl- and methylene- of steroid structure). ^13^C-NMR (CDCl_3_) *δ* (ppm): 35.0 (C1), 30.5 (C2), 71.8 (C3), 36.4 (C4), 42.1 (C5), 27.1 (C6), 26.1 (C7), 36.0 (C8), 33.7 (C9), 34.1 (C10), 28.7 (C11), 73.1 (C12), 46.5 (C13), 48.2 (C14), 23.6 (C15), 27.4 (C16), 47.3 (C17), 12.7 (C18), 23.1 (C19), 35.2 (C20), 17.3 (C21), 31.1 (C22), 30.9 (C23), 173.8 (C24); pyrazine ring: 64.9 (2'-CH_2_), 151.1 (C2'), 145.0 (C3'), 148.8 (C5'), 149.1 (C6'), 21.5 (6'-CH_3_), 21.4 (5'-CH_3_), 20.4 (3'-CH_3_). HRMS (ESI) *m/z*: 527.5201 [M+H]^+^, calcd. for C_32_H_51_N_2_O_4_ 527.3849.

*3α*,*7α*,*12α-Trihydroxy-5β-cholanic acid-3*,*5*,*6-trimethylpyrazin-2-methyl ester* (**6**). Compound **3** (0.350 g, 1.630 mmol) and CA (0.665 g, 1.630 mmol) were dissolved in dry DMF, then K_2_CO_3_ (0.700 g, 5.072 mmol) was added, and the mixture was kept at 85 °C for 4 h. The warm reaction mixture was poured into ice-water and the crude product was extracted with ethyl acetate. After drying the organic layer over anhydrous Na_2_SO_4_ and evaporating the solvent under vacuum, the crude product was purified by flash chromatography with petroleum ether-acetone (5:4) as eluent. White amorphous solid, m.p.: 95.4–96.3 °C, yield 55.4%. ^1^H-NMR (CDCl_3_) *δ* (ppm): 0.67 (s, 3H, H-18), 0.89 (s, 3H, H-19), 0.98 (d, *J* = 5.5 Hz, 3H, H-21), 3.45 (m, 1H, H-3), 3.85 (m, 1H, H-7), 3.97 (m, 1H, H-12), 5.21 (brs, 2H, CH_2_-2'), 2.56 (s, 3H, CH_3_-6'), 2.54 (s, 3H, CH_3_-5'), 2.53 (s, 3H, CH_3_-3'), 1.00–2.50 (27H, methyl- and methylene- of steroid structure). ^13^C-NMR (CDCl_3_) *δ* (ppm): 35.3 (C1), 30.5 (C2), 71.9 (C3), 39.6 (C4), 41.5 (C5), 34.8 (C6), 68.5 (C7), 39.5 (C8), 26.4 (C9), 34.7 (C10), 28.2 (C11), 73.0 (C12), 46.5 (C13), 41.7 (C14), 23.2 (C15), 27.5 (C16), 47.0 (C17), 12.5 (C18), 22.5 (C19), 35.3 (C20), 17.3 (C21), 30.9 (C22), 31.1 (C23), 174.0 (C24); pyrazine ring: 64.9 (2'-CH_2_), 151.1 (C2'), 145.1 (C3'), 148.9 (C5'), 149.2 (C6'), 21.6 (6'-CH_3_), 21.5 (5'-CH_3_), 20.4 (3'-CH_3_). HRMS (ESI) *m/z*: 543.6199 [M+H]^+^, calcd. for C_32_H_51_N_2_O_5_ 543.3798.

*3β-Hydroxyolea-12-en-28-oic acid-3*,*5*,*6-trimethylpyrazin-2-methyl ester* (**7**). Compound **3** (2.103 g, 9.780 mmol) and OA (4.460 g, 9.780 mmol) were dissolved in dry THF, then K_2_CO_3_ (3.500 g, 25.350 mmol) was added and the mixture was refluxed for 1.5 h, filtered and the filtrate was evaporated down under vacuum. The product was separated by flash chromatography with petroleum ether-ethyl acetate (3:2) as eluent and recrystallized from ethyl acetate. White solid, m.p.: 185.9–186.6 °C, yield 57.0%. ^1^H-NMR (CDCl_3_) *δ* (ppm): 0.55, 0.80, 0.90, 0.91, 0.93, 1.00, 1.13 (s, each, 3H, 7×CH_3_), 3.23 (m, 1H, H-3), 2.89 (m, 1H, H-18), 5.26 (brs, 1H, H-12), 5.24, 5.14 (ea, d, *J* = 12.5 Hz, 1H, CH_2_-2'), 2.58 (s, 3H, CH_3_-6'), 2.54 (s, 3H, CH_3_-5'), 2.52 (s, 3H, CH_3_-3'), 1.00–2.50 (23H, methyl- and methylene- of triterpenoid structure). ^13^C-NMR (CDCl_3_) *δ* (ppm): 38.4(C1), 27.2 (C2), 79.0 (C3), 38.8 (C4), 55.2 (C5), 18.3 (C6), 33.1 (C7), 39.2 (C8), 47.6 (C9), 37.0 (C10), 23.7 (C11), 122.5 (C12), 143.6 (C13), 41.7 (C14), 27.6 (C15), 23.1 (C16), 46.9 (C17), 41.3 (C18), 45.9 (C19), 30.7 (C20), 33.9 (C21), 32.7 (C22), 28.1 (C23), 15.6 (C24), 15.3 (C25), 16.8 (C26), 25.9 (C27), 177.2 (C28), 32.4 (C29), 23.4 (C30); pyrazine ring: 64.8 (2'-CH_2_), 150.9 (C2'), 145.5 (C3'), 148.9 (C5'), 149.1 (C6'), 21.6 (6'-CH_3_), 21.4 (5'-CH_3_), 20.5 (3'-CH_3_). HRMS (ESI) *m/z*: 591.6581 [M+H]^+^, calcd. for C_38_H_59_N_2_O_3_ 591.4526.

*3β-Acetoxy-16α-hydroxy-lanosta-8*,*24(31)-diene-21-oic acid-3*,*5*,*6-trimethylpyrazin-2-methyl ester* (**8**) Compound **3** (0.063 g, 0.290 mmol) and PA (0.153 g, 0.290 mmol) were dissolved in dry DMF, then K_2_CO_3_ (0.175 g, 1.270 mmol) was added and the mixture was kept at 85 °C for 1.5 h. The warm reaction mixture was poured into ice-water and the crude product was extracted with ethyl acetate. After drying the organic layer over anhydrous Na_2_SO_4_ and evaporating the solvent under vacuum, the crude products were purified by flash chromatography with petroleum ether-acetone (10:1) as eluent. White amorphous solid, m.p.: 83.2–83.9 °C, yield 45.2%. ^1^H-NMR (CDCl_3_) δ (ppm): 0.71 (s, 3H, H-18), 0.90 (s, 3H, H-28), 0.90 (s, 3H, H-29), 0.99 (s, 3H, H-19), 0.99, 0.97 (ea, d, *J* = 7.0 Hz, 3H, H-26, H-27), 1.11 (s, 3H, H-30), 2.07 (s, 3H, H-33), 4.12 (dd, 1H, H-16), 4.51 (dd, 1H, H-3), 4.67 (s, 1H, H-31), 4.75 (s, 1H, H-31), 5.28, 5.18 (ea, d, *J* = 12.5 Hz, 1H, CH_2_-2'), 2.59 (s, 3H, CH_3_-6'), 2.53 (s, 3H, CH_3_-5'), 2.51 (s, 3H, CH_3_-3'), 1.00–2.50 (23H, methyl- and methylene- of triterpenoid structure). ^13^C-NMR (CDCl_3_) *δ* (ppm): 35.2 (C1), 24.1 (C2), 80.8 (C3), 37.8 (C4), 50.4 (C5), 18.0 (C6), 26.4 (C7), 134.2 (C8), 134.3 (C9), 36.9 (C10), 20.5 (C11), 28.9 (C12), 46.0 (C13), 48.1 (C14), 42.7 (C15), 77.0 (C16), 57.0 (C17), 17.5 (C18), 19.2 (C19), 46.9 (C20), 175.4 (C21), 30.7 (C22), 32.2 (C23), 155.0 (C24), 33.7 (C25), 21.8 (C26), 21.7 (C27), 27.9 (C28), 16.6 (C29), 25.2 (C30), 106.9 (C31), 171.0 (C32), 21.3 (C33); pyrazine ring: 64.6 (2'-CH_2_), 151.2 (C2'), 144.9 (C3'), 149.0 (C5'), 149.1 (C6'), 21.7 (6'-CH_3_), 21.4 (5'-CH_3_), 20.6 (3'-CH_3_). HRMS (ESI) *m/z*: 663.5332 [M + H]^+^, calcd. for C_41_H_63_N_2_O_5_ 663.4737.

*3β-Acetoxy-16α-hydroxy-lanosta-7*,*9(11)*,*24(31)-trien-21-oic acid-3*,*5*,*6-trimethylpyrazin-2-methyl ester* (**9**). Compound **3** (0.062 g, 0.282 mmol) and DPA (0.148 g, 0.282 mmol) were dissolved in dry DMF, then K_2_CO_3_ (0.152 g, 1.101 mmol) was added and the mixture was kept at 85 °C for 1.5 h. The warm reaction mixture was poured into ice-water and the crude product was extracted with ethyl acetate. After drying the organic layer over anhydrous Na_2_SO_4_ and evaporating the solvent under vacuum, the crude products were purified by flash chromatography with petroleum ether-acetone (10:1) as eluent. White amorphous solid, m.p.: 84.9–85.7 °C, yield 42.7%. ^1^H-NMR (CDCl_3_) *δ* (ppm): 0.58 (s, 3H, H-18), 0.91 (s, 3H, H-28), 0.97 (s, 3H, H-29), 0.99 (s, 3H, H-19), 0.99, 0.97 (ea, d, *J* = 6.5 Hz, 3H, H-26, H-27), 1.08 (s, 3H, H-30), 2.08 (s, 3H, H-33), 4.11 (dd, 1H, H-16), 4.52 (dd, 1H, H-3), 4.68 (s, 1H, H-31), 4.75 (s, 1H, H-31), 5.25 (d, 1H, H-11), 5.45 (d, 1H, H-7). 5.25 (q, 2H, CH_2_-2'), 2.60 (s, 3H, CH_3_-6'), 2.53 (s, 3H, CH_3_-5'), 2.50 (s, 3H, CH_3_-3'). 1.00–2.50 (19H, methyl- and methylene- of triterpenoid structure). ^13^C-NMR (CDCl_3_) *δ* (ppm): 35.4 (C1), 24.2 (C2), 80.7 (C3), 37.6 (C4), 49.2 (C5), 22.8 (C6), 120.7 (C7), 141.7 (C8), 145.3 (C9), 37.3 (C10), 116.1 (C11), 35.4 (C12), 44.7 (C13), 48.6 (C14), 43.5 (C15), 76.9 (C16), 57.2 (C17), 17.3 (C18), 22.8 (C19), 46.7 (C20), 175.3 (C21), 30.6 (C22), 32.2 (C23), 155.0 (C24), 33.7 (C25), 21.8 (C26), 21.7 (C27), 28.1 (C28), 17.0(C29), 26.1(C30), 106.9 (C31), 170.9 (C32), 21.3 (C33); pyrazine ring: 64.5 (2'-CH_2_), 151.2 (C2'), 144.9 (C3'), 149.1 (C5'), 148.9 (C6'), 21.7 (6'-CH_3_), 21.4 (5'-CH_3_), 20.6 (3'-CH_3_). HRMS (ESI) *m/z*: 661.5009 [M+H]^+^, calcd. for C_41_H_61_N_2_O_5_ 661.4580.

*(3β*,*18α*,*20β)-3-Hydroxy-11-oxoolean-12-en-29-oicacid-3*,*5*,*6-trimethylpyrazin-2-methyl ester* (**10**). Compound **3** (0.102 g, 0.475 mmol) and GA (0.223 g, 0.475 mmol) were dissolved in dry DMF, then K_2_CO_3_ (0.237 g, 1.715 mmol) was added and the mixture was kept at 85 °C for 2.5 h. The warm reaction mixture was poured into ice-water and the crude product was extracted with ethyl acetate. After drying the organic layer over anhydrous Na_2_SO_4_ and evaporating the solvent under vacuum, the crude product was purified by flash chromatography with petroleum ether-acetone (10:1) as eluent and recrystallized from acetone. Colorless crystals, m.p.: 225.7–226.5 °C, yield 53.0%. ^1^H-NMR (CDCl_3_) *δ* (ppm): 0.81, 0.82, 1.01, 1.13, 1.14, 1.20, 1.36 (s, each, 3H, 7 × CH_3_), 3.23 (m, 1H, H-3), 5.55 (s, 1H, H-12), 5.27, 5.19 (ea, d, *J* = 12.5 Hz, 1H, CH_2_-2'), 2.55 (s, 3H, CH_3_-6'), 2.54 (s, 3H, CH_3_-5'), 2.52 (s, 3H, CH_3_-3'), 1.00–3.00 (22H, methyl- and methylene- of triterpenoid structure). ^13^C-NMR (CDCl_3_) *δ* (ppm): 39.2 (C1), 27.3 (C2), 78.8 (C3), 39.2 (C4), 55.0 (C5), 17.5 (C6), 32.8 (C7), 43.2 (C8), 61.8 (C9), 37.1 (C10), 200.0 (C11), 128.6 (C12), 168.9 (C13), 45.4 (C14), 26.5 (C15), 26.4 (C16), 31.9 (C17), 48.0 (C18), 41.1 (C19), 44.2 (C20), 31.2 (C21), 37.7 (C22), 28.5 (C23), 15.6 (C24), 16.4 (C25), 18.7 (C26), 23.4 (C27), 28.1 (C28), 28.5 (C29), 176.1 (C30); pyrazine ring: 64.7 (2'-CH_2_), 151.1 (C2'), 145.0 (C3'), 148.4 (C5'), 149.3 (C6'), 21.5 (6'-CH_3_), 21.5 (5'-CH_3_), 20.4 (3'-CH_3_). HRMS (ESI) *m/z*: 605.5377 [M+H]^+^, calcd. for C_38_H_57_N_2_O_4_ 605.4318.

*3-Phenyl-2-propenoic acid-3*,*5*,*6-trimethylpyrazin-2-methyl ester* (**11**). Compound **3** (0.701 g, 3.260 mmol) and CIA (0.482 g, 3.260 mmol) were dissolved in dry DMF, then K_2_CO_3_ (0.561 g, 4.056 mmol) was added and the mixture was kept at 85 °C for 2.0 h. The warm reaction mixture was poured into ice-water and the crude product was extracted with ethyl acetate. After drying the organic layer over anhydrous Na_2_SO_4_ and evaporating the solvent under vacuum, the crude product was puriﬁed by flash chromatography with petroleum ether-ethyl acetate (4:1) as eluent. White solid, m.p.: 57.7–58.4 °C, yield 61.3%. ^1^H-NMR (CDCl_3_) *δ* (ppm): 7.53–7.54 (m, 2H, H-5, 9), 7.40 (m, 3H, H-6, 7, 8), 6.51 (d, *J* = 16 Hz, 1H, H-2), 7.75 (d, *J* = 16 Hz, 1H, H-3), 5.36 (s, 2H, CH_2_-2'), 2.60 (s, 3H, CH_3_-6'), 2.55 (s, 3H, CH_3_-5'), 2.54 (s, 3H, CH_3_-3'). ^13^C-NMR (CDCl_3_) *δ* (ppm): 166.6 (C1), 117.4 (C2), 145.6 (C3), 134.3 (C4), 128.2 (C5, 9), 130.5 (C6, 8), 128.9 (C7); pyrazine ring: 65.2 (2'-CH_2_), 151.3 (C2'), 144.9 (C3'), 149.1 (C5'), 149.2 (C6'), 21.7 (6'-CH_3_), 21.6 (5'-CH_3_), 20.5(3'-CH_3_). HRMS (ESI) *m/z*: 283.2580 [M+H]^+^, calcd. for C_17_H_19_N_2_O_2_ 283.1447.

### 3.2. Bio-Evaluation Methods

#### 3.2.1. Cytotoxicity Evaluation

The cytotoxicities of these compounds were tested on five cancer cell lines by the standard MTT assay. The human cancer cells lines HCT-8, Bel-7402, BGC-823, A-549, A2780 were provided by the Chinese Academy of Medical Sciences & Peking Union Medical College. The growing tumor cells at a density 10^4^ cells/mL were exposed to various concentrations of the tested drugs and incubated in a 96-well microtiter plate for 96 h (37 °C, 5% CO_2_). After MTT solution (20 μL, 5 mg/mL) was added to each well, the plate was incubated for a further 4 h. Then the media was removed. Formazan crystals were dissolved with DMSO (150 μL). After mixing well, the absorbance was quantified at 570 nm with a BIORAD 550 spectrophotometer. Wells containing no drugs were used as blanks. The IC_50_ values were defined as the concentration of compounds that produced a 50% reduction of surviving cells and calculated using Logit-method. Tumor cell growth inhibitory rate was calculated in the following equation (1):

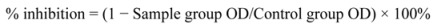
(1)


#### 3.2.2. Angiogenesis Assay

Fertilized White Leghorn chicken eggs, provided by the Chinese Academy of Agricultural Sciences, were placed in an incubator as soon as embryogenesis started and were kept under constant humidity of 65% at 37 °C. On day 7, a square window was opened on the shell and physiological saline (0.1 mL) was injected in to detach the shell membrane. Then gelatin sponges carrying the TMP stimulator derivatives at 10 μg/egg and 40 μg/egg were implanted, respectively. The control group was treated with physiological saline. The windows were sealed with medical adhesive tape and the incubation went on till the experiment day. The above steps were performed under sterile conditions. On the 11th day, the tapes were removed and the entire CAM was detached after tissue fixation with methanol/acetone (1:1, v/v). Then we used computer-assisted image tracking to obtain absolute values for the number of microvessels which were 1 to 100 μm in diameter. Data were analyzed using the *t*-test option of the of Statistics Analysis System, the values were expressed as mean ± s of 6 observations and *P* < 0.05 was considered significant.

#### 3.2.3. Acute Toxicity

Kunming mice (Beijing Vital River Laboratory Animal Technology Company Limited, China) of both sexes, weighing 18–22 g, were divided into four groups of 10 animals matched in weight and size. The mice were placed in cages and kept under standard environmental conditions with a standard rodent diet and water *ad libitum* under a 12 h light-dark cycle. They were deprived of food for 24 h but allowed free access to tap water throughout the experiments. This research was carried out in accordance with the “Regulation for the Administration of Affairs Concerning Experimental Animals” (State Council of China, 1988).

The maximum suspended dose (75 mg/mL) of **5** was prepared in bean oil solution, then one group of 20 mice of both sexes were administered the maximum tolerated dose (0.4 mL/10 g) by oral administration. The other 20 mice, the control group, were gave bean oil (0.4 mL/10 g) via gavage.The general behavior of the mice was observed continuously for 1 h after the treatment and then intermittently for 4 h, and thereafter over a period of 24 h. The mice were further observed for up to 14 days following treatment for any signs of toxicity and deaths, and the latency of death. Behavioral, toxic effects and mortality response were recorded.

## 4. Conclusions

A series of TMP derivatives were synthesized through conjugation of anti-tumor bioactive compounds via ester or ether bonds. All the target compounds were obtained in three steps and the route was much easier than that used in a previous report [26]. As the starting materials showed broad anti-tumor activities, we chose five different human cancer cell lines to evaluate the TMP derivatives. Compounds **4** and **5** not only inhibited proliferation of all cancer cells but also dramatically suppressed new angiogenesis in CAM. In addition, the acute toxicity assay of compound **5** indicates no toxicity. Based on the studies of pharmacology and acute toxicity, compounds **4** and **5** represent new hits for anticancer drug discovery and development. The results suggest that the attempt to apply structure combination to discover more efficient, low toxicity and multi-effective anti-tumor lead compounds from TCM formulations is viable. Our completed work lays the foundation for further research on the anti-tumor mechanism of TMP derivatives. 

## Supplementary Materials

Supplementary materials can be accessed at: http://www.mdpi.com/1420-3049/17/5/4972/s1.

## References

[1] Fialho A.M., Gupta T.K.D., Chakrabarty A.M. (2007). Designing promiscuous drugs? Look at what nature made. Lett. Drug Des. Discov..

[2] Wu D.H., Xu X.J. (2011). A new practice: Study on the molecular mechanism of traditional Chinese medicine by computational pharmacology methods: Part 1: Pharmacokinetic modeling and chemical space distribution. Lett. Drug Des. Discov..

[3] Wu D.H., Xu X.J., Zhang M.Z., Wang L. (2011). A new practice: Study on the molecular mechanism of traditional Chinese medicine by computational pharmacology methods: Part 2: Pharmacodynamic modeling and distribution on ligand-target space of effective components. Lett. Drug Des. Discov..

[4] Efferth T., Li P.C., Konkimalla V.S., Kaina B. (2007). From traditional Chinese medicine to rational cancer therapy. Trends Mol. Med..

[5] Wang C., Cao B., Liu Q.Q., Zou Z.Q., Liang Z.A., Gu L., Dong J.P., Liang L.R., Li X.W., Hu K. (2011). Oseltamivir compared with the Chinese traditional therapy maxingshigan-yinqiaosan in the treatment of H1N1 influenza: A randomized trial. Ann. Intern. Med..

[6] Dennis N. (2003). The new face of traditional Chinese medicine. Science.

[7] Zhang J.L., Wang H., Pi H.F., Ruan H.L., Zhang P., Wu J.Z. (2009). Structural analysis and antitussive evaluation of five novel esters of verticinone and bile acids. Steroids.

[8] Zhang J.L., Wang H., Chen C., Pi H.F., Raun H.L., Zhang P., Wu J.Z. (2009). Addictive evaluation of cholic acid-verticinone ester, a potential cough therapeutic agent with agonist action of opioid receptor. Acta Pharmacol. Sin..

[9] Hao Y.Z., Wang P.L., Hong Y., Lei H.M. (2010). Synthesis and structure identification of tetramethylpyrazine-protocatechuic acid and effects on hypoxic-ischemic brain damage. Pharmacol. Clin. Chin. Mater. Med..

[10] Ni M., Chen Z.S., Peng Y.H. (2005). Clinical study of intraperitoneal chemotherapy plus Shiquandabu drink on advanced gastric cancer. Chin. Clin. Oncol..

[11] Guo G., Xu J.H. (2011). Study of Shiquandabu decoction’s effecting on inhibit tumor invasion and metastasis. Glob. Tradit. Chin. Med..

[12] Yin J., Yu C., Yang Z., He J.L., Chen W.J., Liu H.Z., Li W.M., Liu H.T., Wang Y.X. (2011). Tetramethylpyrazine inhibits migration of SKOV3 human ovarian carcinoma cells and decreases the expression of interleukin-8 via the ERK1/2, p38 and AP-1 signaling pathways. Oncol. Rep..

[13] Kunnumakkara A.B., Anand P., Aggarwal B.B. (2008). Curcumin inhibits proliferation, invasion, angiogenesis and metastasis of different cancers through interaction with multiple cell signaling proteins. Cancer Lett..

[14] Bill M.A., Bakan C., Benson D.M.J., Fuchs J., Young G., Lesinski G.B. (2009). Curcumin induces proapoptotic effects against human melanoma cells and modulates the cellular response to immunotherapeutic cytokines. Mol. Cancer Ther..

[15] Martinez J.D., Stratagoules E.D., LaRue J.M., Powell A.A., Gause P.R., Craven M.T., Payne C.M., Powell M.B., Gerner E.W., Earnest D.L. (1998). Different bile acids exhibit distinct biological effects: The tumor promoter deoxycholic acid induces apoptosis and the chemopreventive agent ursodeoxycholic acid inhibits cell proliferation. Nutr. Cancer.

[16] Wu Z., Lü Y., Wang B., Liu C., Wang Z.R. (2003). Effects of bile acids on proliferation and ultrastructural alteration of pancreatic cancer cell lines. World J. Gastroenterol..

[17] Zhang P.X., Li H.M., Chen D., Ni J.H., Kang Y.M., Wang S.Q. (2007). Oleanolic acid induces apoptosis in human leukemia cells through caspase activation and poly (ADP-ribose) polymerase cleavage. Acta Bioch. Bioph. Sin..

[18] Gapter L., Wang Z., Glinski J., Ng K.Y. (2005). Induction of apoptosis in prostate cancer cells by pachymic acid from Poria cocos. Biochem. Biophys. Res. Commun..

[19] Li G., Xu M.L., Lee C.S., Woo M.H., Chang H.W., Son J.K. (2004). Cytotoxicity and DNA topoisomerases inhibitory activity of constituents from the sclerotium of *Poria cocos*. Arch. Pharm. Res..

[20] Ekmekcioglu C., Feyertag J., Marktl W. (1998). Cinnamic acid inhibits proliferation and modulates brush border membrane enzyme activities in Caco-2 cells. Cancer Lett..

[21] Abe H., Ohya N., Yamamoto K.F., Shibuya T., Arichi S., Odashima S. (1987). Effects of glycyrrhizin and glycyrrhetinic acid on growth and melanogenesis in cultured B16 melanoma cells. Eur.J. Cancer Clin. Oncol..

[22] Ding M.Y., Luo S.Z., Liu H., Chen P.R., Liu D.L. (2000). Determination of tetramethylpyrazine in animal serum and cerebrospinal fluid by high performance liquid chromatography. Chin. J. Chrom..

[23] Li Q.Y., Gao Y., Qiu W., Zu Y.G., Su L., He W.N., Deng X.Q. (2011). Synthesis and anti-tumor activity of novel camptothecin-bile acid analogues. Lett. Drug Des. Discov..

[24] Lei H.M., Li Q., Wang P.L. (2011). Synthesis and Application of Anticancer Agents (LQC-Y).

[25] Lei H.M., Li Q., Wang P.L. (2011). Preparation and Application of Agents (LQC-X) for Treatment Ischemic Brain Injury and Its Sequelae.

[26] Liu X.Y., Zhang R., Xu W.F., Li C.W., Zhao Q.Q., Wang X.P. (2003). Synthesis of the novel liqustrazine derivatives and their protective effect on injured vascular endothelial cell damaged by hydrogen peroxide. Bioorg. Med. Chem. Lett..

[27] Tai T., Shingu T., Kikuchi T., Tezuka Y., Akahori A. (1995). Isolation of lanostane-type triterpene acids having an acetoxyl group from sclerotia of *Poria cocos*. Phytochemistry.

[28] Folkman J. (1971). Tumor angiogenesis: Therapeutic implications. N. Engl. J. Med..

[29] Shaked Y., Henke E., Roodhart J.M., Mancuso P., Langenberg M.H., Colleoni M., Daenen L.G., Man S., Xu P., Emmenegger U. (2008). Rapid chemotherapy-induced acute endothelial progenitor cell mobilization: implications for antiangiogenic drugs as chemosensitizing agents. Cancer Cell.

[30] Fotsis T., Zhang Y.M., Pepper M.S., Adlercreutz H., Montesano R., Nawroth P.P., Schweigerer L. (1994). The endogenous oestrogen metabolite 2-methoxyestradiol inhibits angiogenesis and suppresses tumor growth. Nature.

[31] Luo Y., Jiang F., Cole T.B., Hradil V.P., Reuter D., Chakravartty A., Albert D.H., Davidsen S.K., Cox B.F., McKeegan E.M. (2012). A novel multi-targeted tyrosine kinase inhibitor, linifanib (ABT-869), produces functional and structural changes in tumor vasculature in an orthotopic rat glioma model. Cancer Chemother. Pharmacol..

[32] El-Azab M., Hishe H., Moustafa Y., El-Awady E.-S. (2011). Anti-angiogenic effect of resveratrol or curcumin in Ehrlich ascites carcinoma-bearing mice. Eur. J. Pharmacol..

[33] Lee D.Y., Kim S.K., Kim Y.S., Son D.H., Nam J.H., Kim I.S., Park R.W., Kim S.Y., Byun Y. (2007). Suppression of angiogenesis and tumor growth by orally active deoxycholic acid-heparin conjugate. J. Control. Release.

[34] Li H.Q., Wu X.Z., Bai D., Yang Y.N., Lei H.M. (2011). Screening active fraction of compound Sanhuang capsules for inhibition of angiogenesis in tumor. Chin. J. Exp. Tradit. Med. Formulae.

[35] O’Neill V.J., Twelves C.J. (2002). Oral cancer treatment: Developments in chemotherapy and beyond. Br. J. Cancer.

[36] Garcea G., Jones D.J., Singh R., Dennison A.R., Farmer P.B., Sharma R.A., Steward W.P., Gescher A.J., Berry D.P. (2004). Detection of curcumin and its metabolites in hepatic tissue and portal blood of patients following oral administration. Br. J. Cancer.

[37] Cheng A.L., Hsu C.H., Lin J.K., Hsu M.M., Ho Y.F., Shen T.S., Ko J.Y., Lin J.T., Lin B.R., Ming-Shiang W. (2001). Phase I clinical trial of curcumin, a chemopreventive agent, in patients with high-risk or pre-malignant lesions. Anticancer Res..

[38] Dhillon N., Wolf R.A., Abbruzzese J.L., Hong D.S., Camacho L.H., Li L., Braiteh F.S., Kurzrock R. (2006). Phase II clinical trial of curcumin in patients with advanced pancreatic cancer. J. Clin. Oncol..

