# Supplementary material for: Synthesis and Biological Evaluation of New Ligustrazine Derivatives as Anti-Tumor Agents
Source: Molecules. 2012 Apr 30;17(5):4972–85. doi: 10.3390/molecules17054972 (PMC6268357; doi:10.3390/molecules17054972)

Supporting Information

ESI mass SPECTRA of Compound **4**

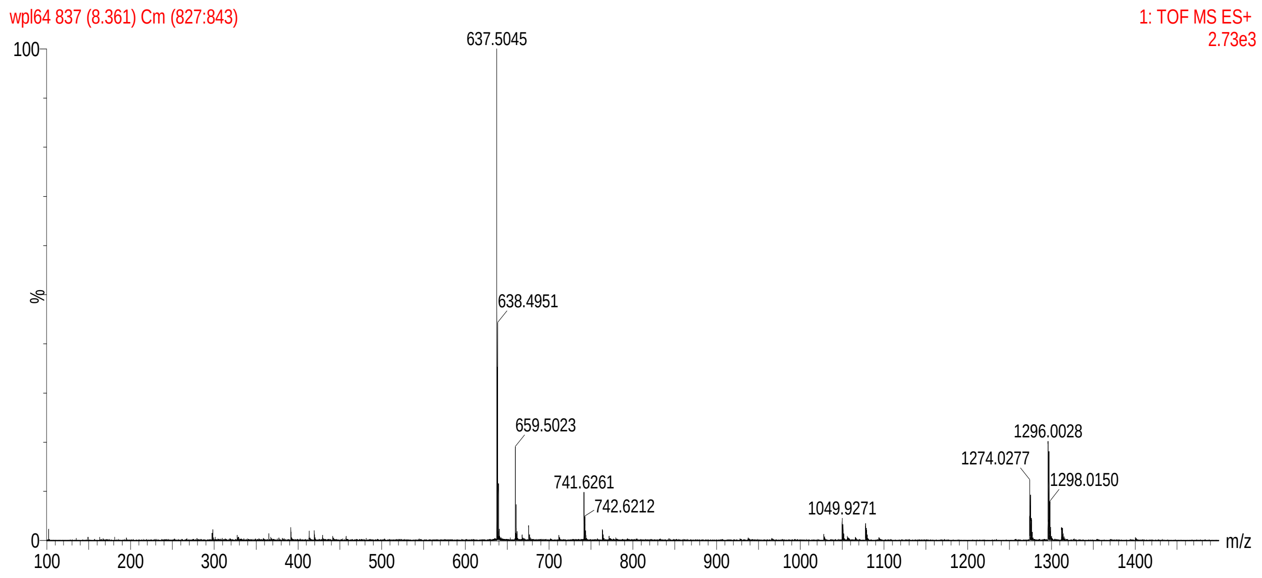

ESI mass SPECTRA of Compound **5**

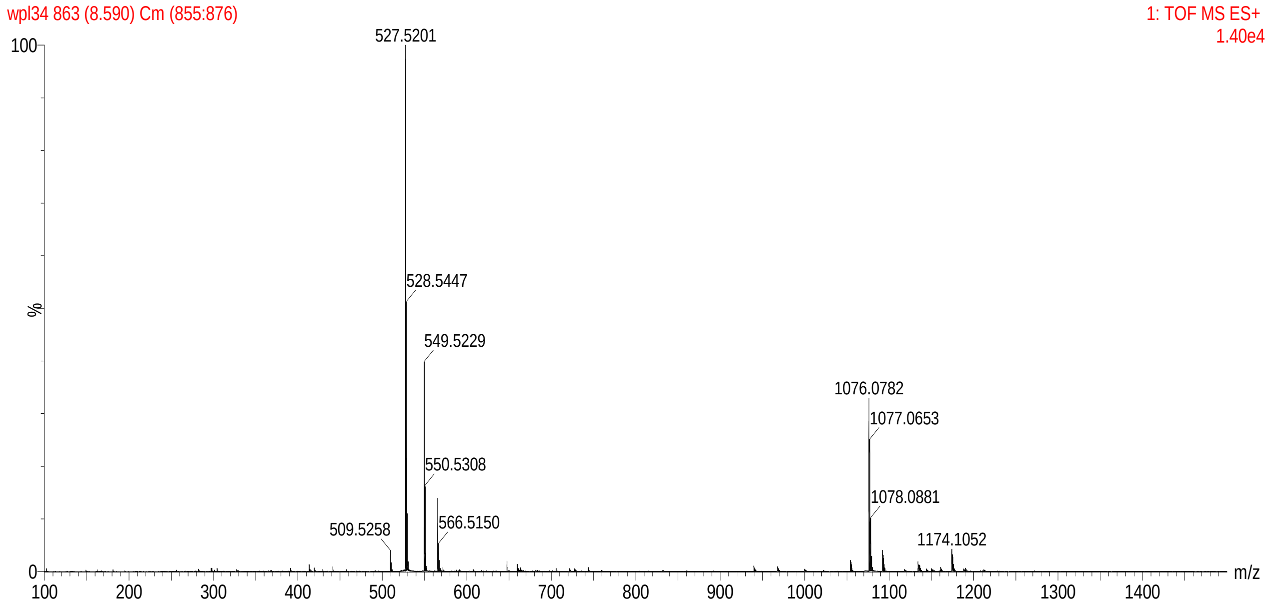

^{1}H-NMR spectrum ($CDCl_3$) of compound **4**

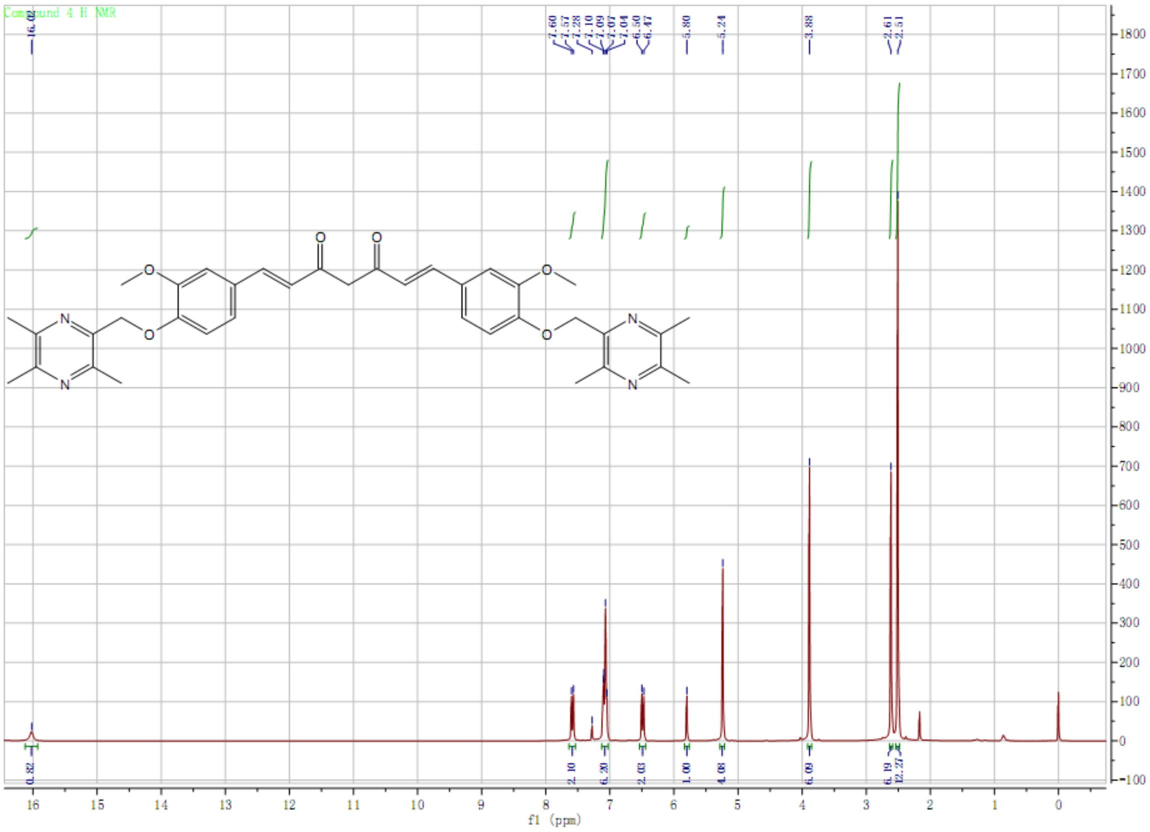

^{13}C-NMR spectrum ($CDCl_3$) of compound **4**

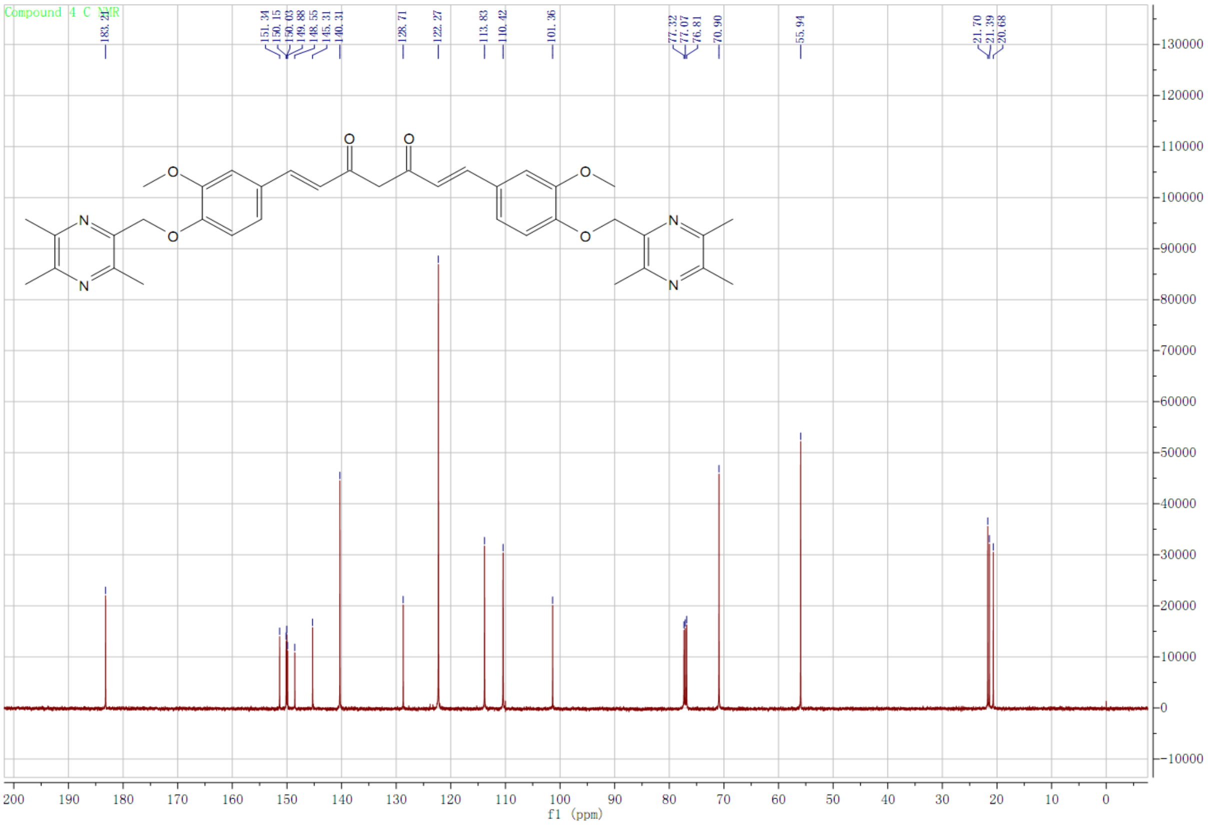

^{1}H-NMR spectrum ($CDCl_3$) of compound **5**

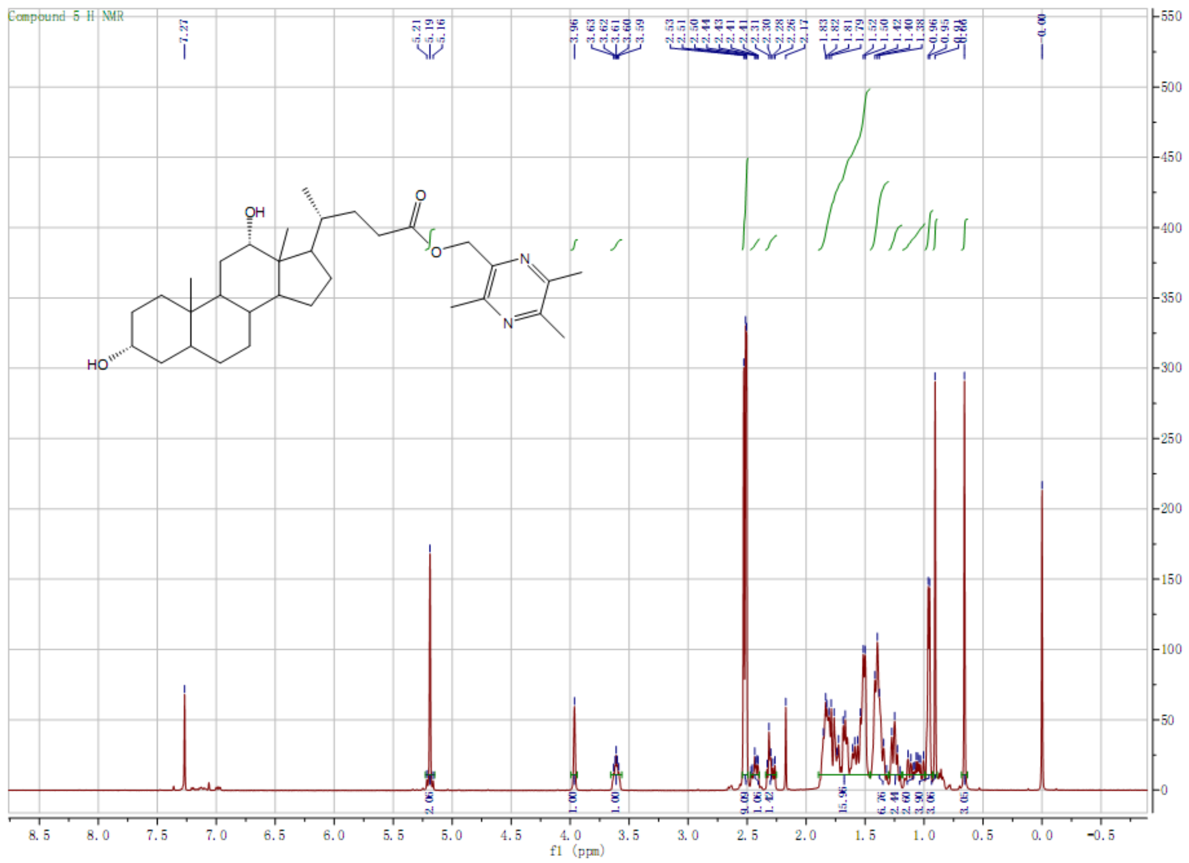

^{13}C-NMR spectrum ($CDCl_3$) of compound **5**

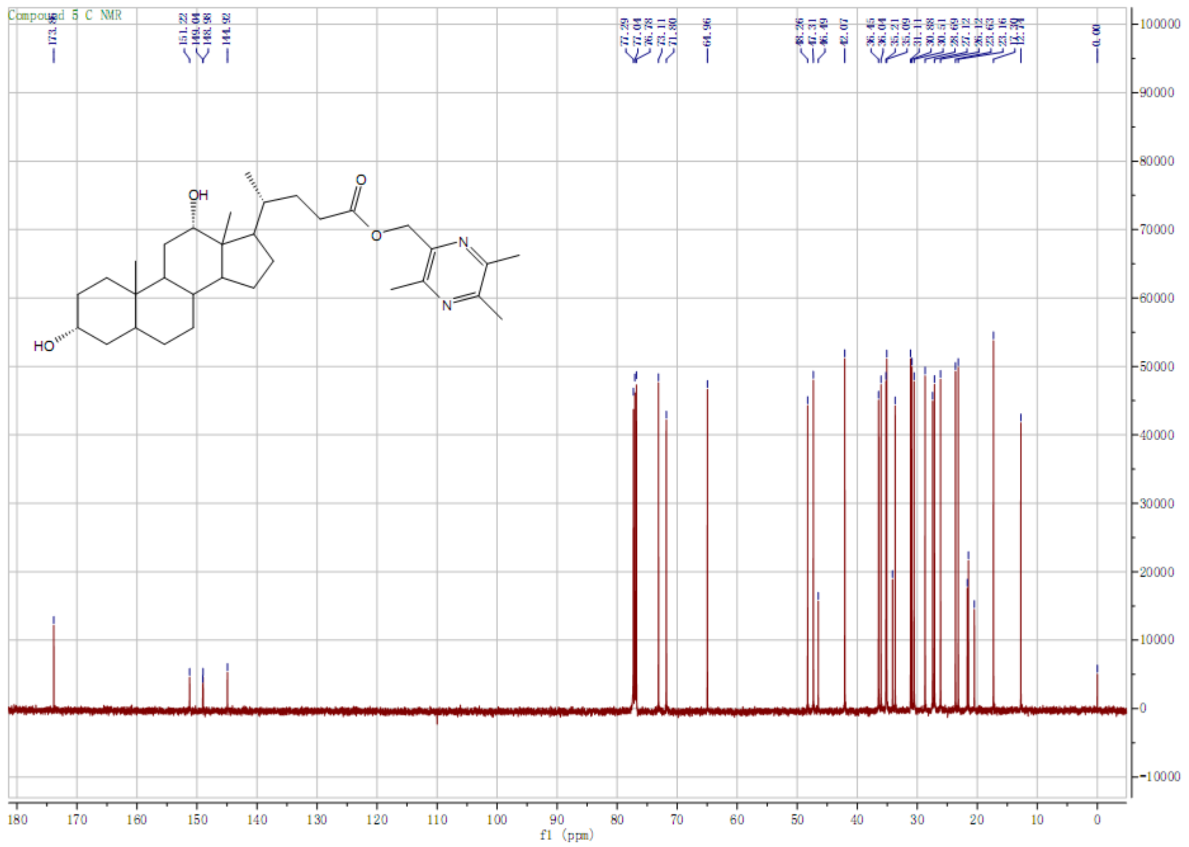

DEPT spectrum ($CDCl_3$) of compound **7**

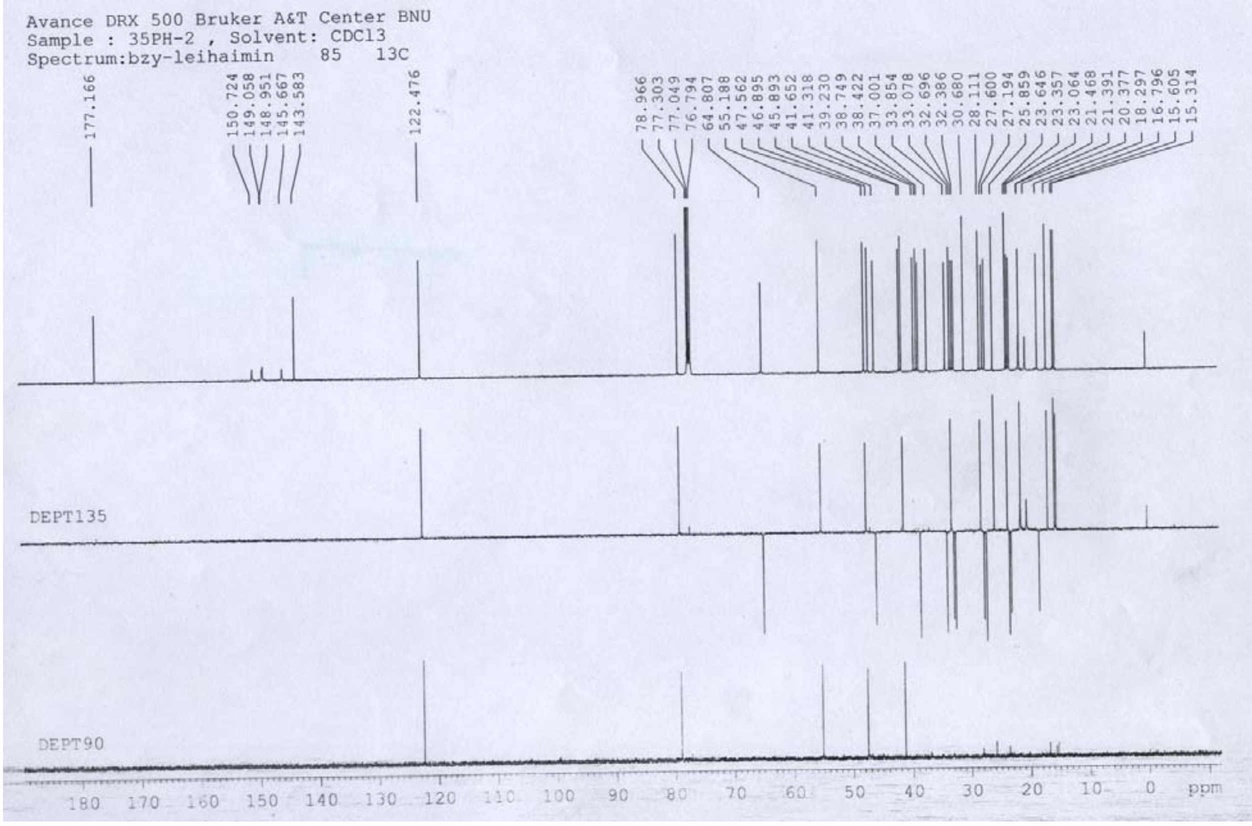

^1H–^1HCOSY spectrum ($CDCl_3$) of compound **7**

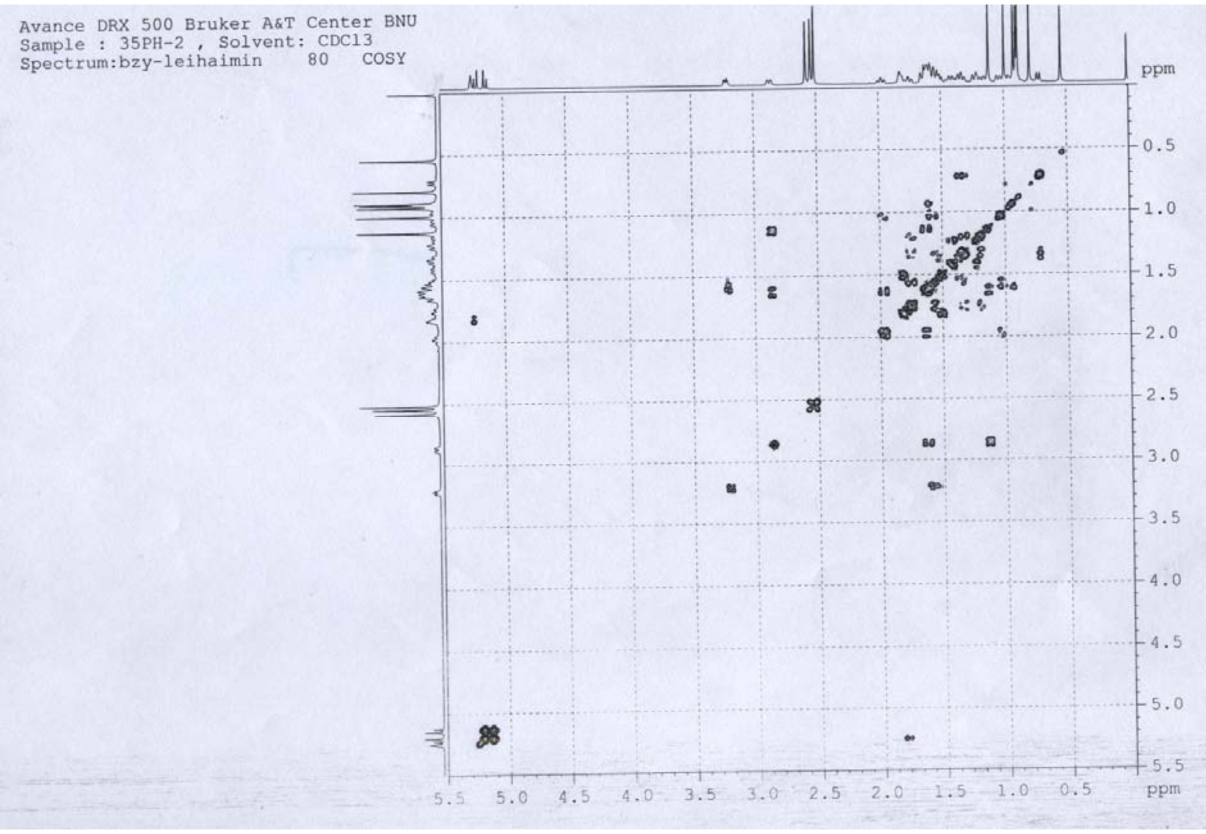

HMBC spectrum ($CDCl_3$) of compound **7**

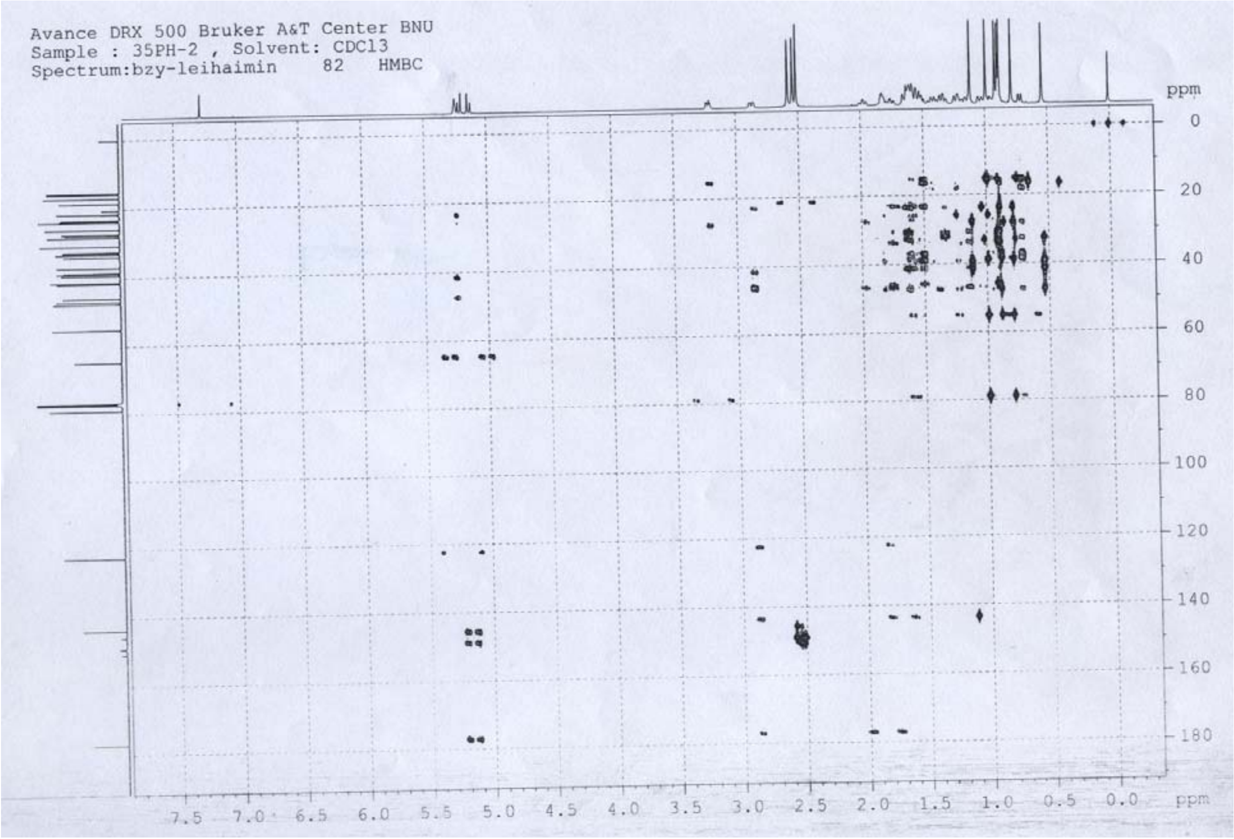

HSQC spectrum ($CDCl_3$) of compound **7**

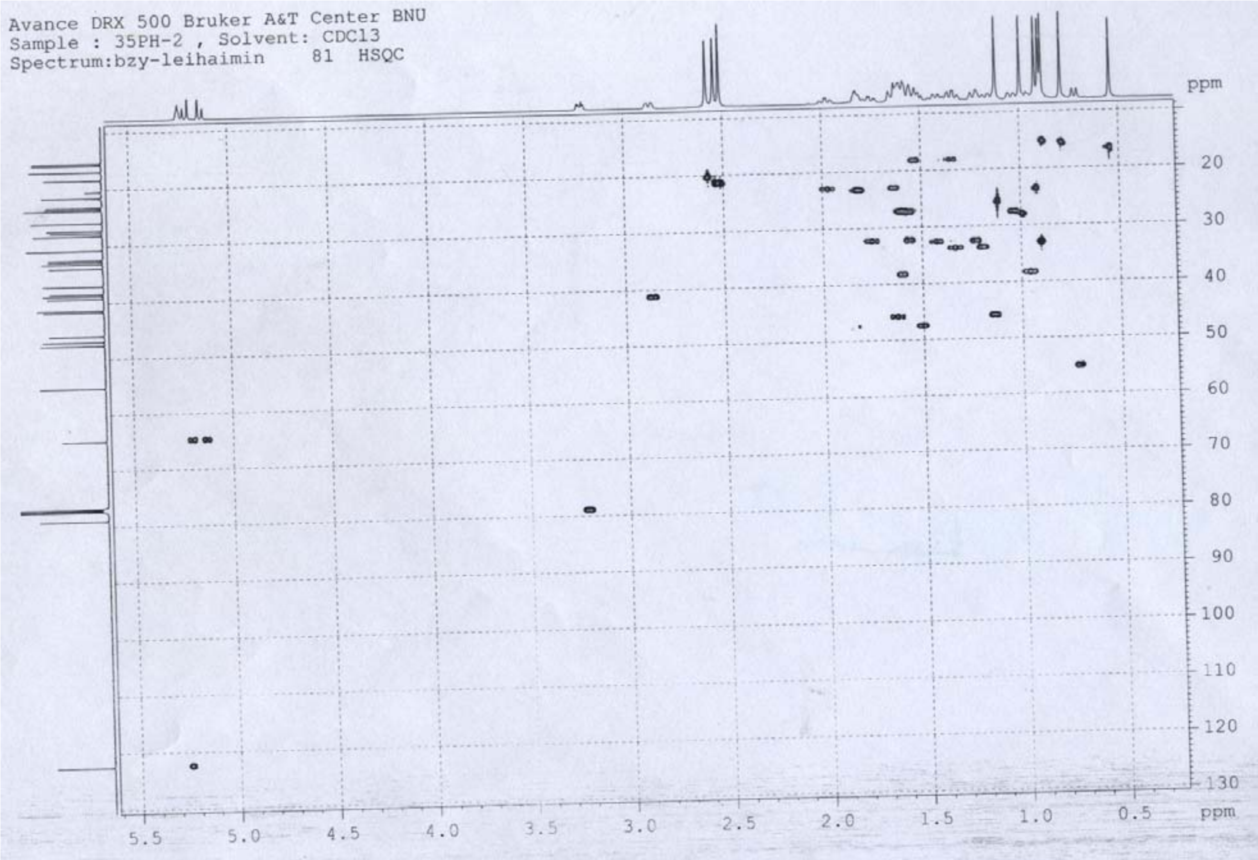

ROESY spectrum ($CDCl_3$) of compound **7**

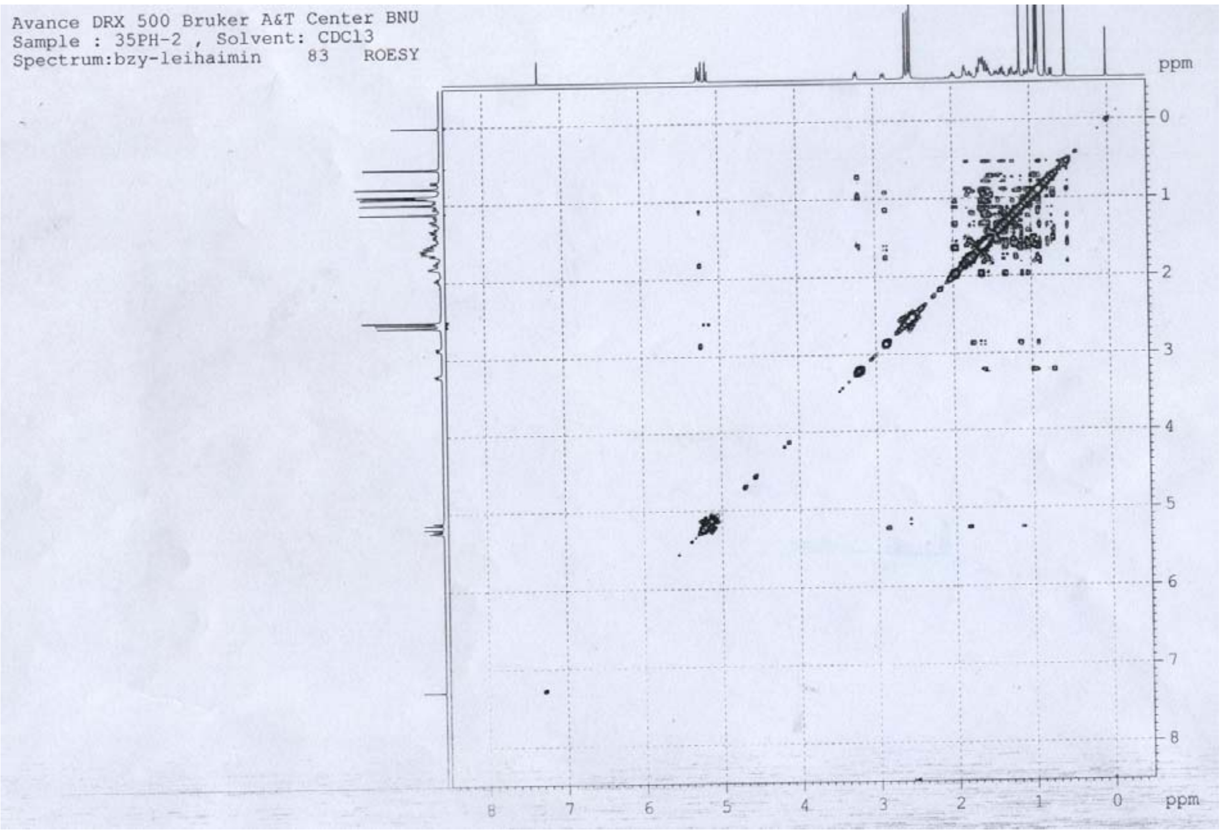

Supplement: Supplementary file 1 [file molecules-17-04972-s001.pdf]
